# Is ‘Long Covid’ similar to ‘Long SARS’?

**DOI:** 10.1093/oxfimm/iqac002

**Published:** 2022-06-09

**Authors:** John Patcai

**Affiliations:** Division of Physiatry, Department of Medicine, University of Toronto, Toronto, ON, Canada; Department of Medicine, McMaster University, Hamilton, ON L8N 3Z5, Canada

## BACKGROUND

### Severe acute respiratory syndrome

In 2002–03, a Severe Acute Respiratory Syndrome (SARS) coronavirus caused a pandemic. It was described as a novel virus, meaning that it seemed to be unrelated to other viruses directly. Worldwide there were approximately 8000 cases and over 800 deaths. Toronto (Ontario, Canada) had the largest outbreak outside of Asia, with 251 cases and 41 deaths, with health care workers making up 43% of the cases [1].

### Covid

The World Health Organisation (WHO) has recorded about 500 million Covid-19 cases and 6 million deaths globally, up to mid-April 2022 [2]. How many people have suffered from Long Covid [also called post acute sequelae of COVID-19 (PASC)]? We have both too much evidence and insufficient evidence. There are many, many articles published. There is incomplete agreement as to criteria for inclusion, symptoms, severity of symptoms and length of time symptoms have persisted. There is the question of what proof of Covid is required (is a self-reported test adequate?) and whether the study setting is in the community or whether it is post hospitalization. In the UK, the official register provides a prevalence of ongoing post-Covid symptoms at about 8% of cases (1.8 million people [3] post 22.3 million cases [4]). A recent Lancet preprint [5] (i.e. preliminary, not yet accepted for publication and without peer review) systematic review and meta-analysis including 196 studies and 120,970 participants showed that long COVID may affect more than half of the patients, after a median of 6 months from the diagnosis. It is expected that with time, the exact numbers will become more clear. However, it is now already clear that the numbers are very significant. To deal with those staggering numbers of people with ongoing Long Covid symptoms, innumerable rehabilitation programs have sprung up. However, since Long Covid is new, there is no knowledge as to what:

- Makes a good rehab program for this population;
- What is cost-effective;
- What services are needed and helpful;
- What are the short-term and long-term outcomes with and without rehabilitation?

These questions cannot yet be answered. However, if as seems likely, Long Covid is similar to the long-term outcomes post SARS, then predictions can be made. Since the term ‘Long Covid’ seems to have taken hold, I will retrospectively refer to the collective symptoms post 2003 as ‘Long SARS’. It should be noted that all the Long SARS patients in my experience were ‘severe’, as all our patients were very sick, hospitalized and many went through the ICU. The literature on Long Covid includes all levels of severity from asymptomatic to fatal. Severity of illness has not yet been established as a risk for Long Covid but it remains as a possibility.

## REHABILITATION

In 2004, our hospital [6] created an extensive interdisciplinary rehabilitation program for 50 severely impaired post-SARS patients. At the time I was the Medical Director and the Chief of Staff. The intensity and duration of the program, together with the number of service providers, is unlikely to be matched by any current program. Ours was the gold standard of rehab programs for post coronavirus symptomatology. It was funded by the Workplace Safety and Insurance Board (WSIB) of Ontario exclusively as a treatment program. There was no possibility of this being funded as a research program. The WSIB case coordinators were generally quite accustomed, as part of their job, to approve or deny treatments in general. They almost never denied any form of treatment to the patients in this particular program and in fact pushed to make treatment as extensive as possible.

The program initially was 3–5 h a day, 3 days a week. The participants ranged in age from the mid-20s to the mid-60s. There was extensive physical rehabilitation and an even larger component focused on cognition and psychological needs. The core treatment team members were the nurse practitioner, occupational therapist, physiotherapist, physiatrist (MD) and psychologist. Core members saw each and every patient. At the recommendation of any of the core team members, the patients could be seen and treated by consulting team members. On site, these included: acupuncturist, chiropractor, dietician, registered massage therapist and pharmacist. Off site, further consulting members included: neuropsychologist, respirologist, cardiologist, psychiatrist and sleep expert (also a psychologist). We had further consultant specialties available in infectious disease, neurology, rheumatology and urology, although they were rarely used. We measured function on a number of scales [Canadian Occupational Performance Measure, Six Minute Walk Test, St. George’s Respiratory Questionnaire, Exercise Testing Modified Bruce Treadmill, SF-36, Cognistat, Fatigue Severity Scale, McGill Pain Questionnaire, SJRH/TRI Outpatient Patient Satisfaction Survey, Beck’s Anxiety Inventory, Spirometry Measures, Pittsburgh Sleep Quality Index, HADS (the hospital anxiety and depression scale), Beck’s Depression inventory, Post Traumatic Stress Disorder Checklist, Civilian Version]. Other tests available to us included PFT (pulmonary function tesing), EMG (electromyography), X-rays, neuroimaging and sleep studies. The main program was phased out before 2007, but a follow-on program was started for the psychological components that required ongoing treatment. Our last publication on psychological outcomes was at the 7-year mark [7].

## SYMPTOMS

The most prevalent Long Covid symptoms currently are reported as being functional mobility impairments, pulmonary abnormalities and mental health disorders but over 200 symptoms, involving 10 body systems have been listed [8]. In our Long SARS rehab program, there were multiple symptoms in multiple systems reported by every patient. There were overlapping commonalities. The most prevalent symptoms were in the areas of fatigue, respiratory system, cognition, mental health and sleep disturbance. Some symptoms were so common that I made a checklist for each patient visit, detailing the intensity of each symptom on a Likert scale (Appendix 1). The symptomatology for Long Covid and Long SARS is very similar, but not very specific.

## REPORTS

I regret that we were not able to get the funding and resources to publish the data from the entire Long SARS rehab program and I suspect that most of that data has now been lost. The following is what is available. These summaries are all from our Long SARS program team members unless other attribution is given.

**Figure 1:**
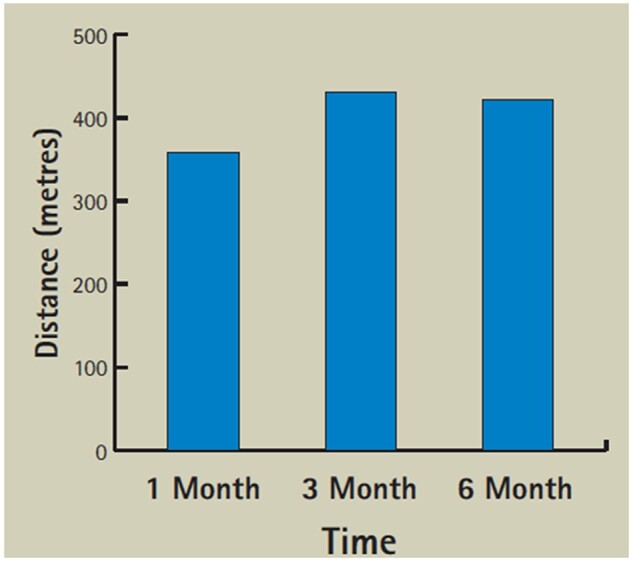
Average 6-min walk result.

**Figure 2:**
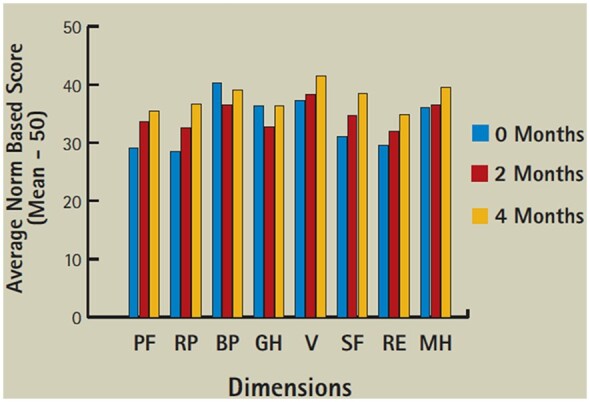
Average SF 36 scores.

A preliminary report appeared to show mild improvement in 6-min walk tests in the first 6 months (Fig. 1) and SF36 [9] in the first 4 months (Fig. 2) [10]. These were not subjected to any statistical analysis.

Our pulmonary function testing was not remarkable and did not warrant statistical analysis. There was initially some weakness of the respiratory muscles, but any small initial abnormality did not persist.

A study [11] (not from our program, but that did come from Toronto, and included some of our patients) found that at 1 year, ‘Lung volume and spirometric measurements were normal by 6 months, but carbon monoxide diffusion capacity remained low throughout the 12-month follow-up. No patients required supplemental oxygen at 12 months, but 6 percent of patients had arterial oxygen saturation values below 88 percent during exercise. The median score for the physical role domain of the Medical Outcomes Study 36-item Short-Form General Health Survey (a health-related quality-of-life measure) increased from 0 at 3 months to 25 at 12 months (score in the normal population, 84). The distance walked in six minutes increased from a median of 281 m at 3 months to 422 m at 12 months; all values were lower than predicted’. In summary, they did find some improvements but overall, there remained significant ongoing impairments in lung function, quality of life and in the ability to walk distances at normal speed.

A poster [12] on the support group process showed that the psychological sequelae of SARS included problems with anxiety, anger, cognition, fear, hypersensitivity, sense of loss and stigma. They found that ‘The Psychological Support Group was shown to be a successful mode of delivery of mental health services. Feedback from the clients was overwhelmingly positive. A psychoeducational group was formed to complement the support group and offer clients concrete tools for coping with the emotional difficulties individuals identified in the support group. Many of the individuals who attended the psychological support group and had an opportunity to discuss their difficulties went on to the psychoeducational group which provided them with tools for coping’.

We published on sleep disturbances during the first few years of the program [13]. The findings included persistent fatigue, diffuse myalgia, weakness, depression, non-restorative sleep, REM-related apneas/hypopneas, elevated sleep EEG cyclical alternating pattern, alpha EEG sleep anomaly, pre- and post-sleep fatigue and post sleep sleepiness. These findings represent unresolved symptoms associated with significantly disturbed sleep.

Two of our psychologists published a literature review of the psychology literature [14] ‘Studies included in our review consistently reported high rates of emotional distress among survivors, persisting for years post infection’. Their findings from the literature search (up to 51 months) were similar to the findings from our own patient group.

Our published neuropsychological testing results [15] showed ‘specific long-term cognitive deficits associated with SARS’.

Our 7-year follow-up study from the psychology side [7] demonstrated depression, anxiety and post-traumatic stress disorder (PTSD) that did not improve over the years. These conditions appeared to worsen with time, but any worsening did not meet the criteria to show statistical significance. There were also symptoms of pain, reduced vitality and reductions in physical, mental and social functioning. These symptoms worsened from years 4 to 7 in a statistically significant fashion.

One extra clinical note: the medical treatment at that time might itself have contributed to PTSD. Our patients were almost all health care workers. Suddenly they became patients. They all were very ill and admitted to hospital, with pneumonia, and difficulty breathing. Some were intubated. Initially, there were no laboratory tests to make a diagnosis of SARS, so the possible diagnoses were ‘suspected SARS’ and ‘probable SARS’. Our patients were all in the latter category. ‘For suspected cases, we took a broad interpretation of the respiratory symptoms in the WHO criteria to include upper and lower tract clinical features. We confirmed a diagnosis of SARS when a patient was known to have contact with someone with SARS, had documented persistent fever (>38°C), a consistent clinical course of the illness, and evidence of pneumonia [16]’. They were legally not allowed to discharge themselves. They were fully isolated, with no contact with friends or family. All possible materials in the room were discarded after cleaning, including the destruction of the telephones. These health care workers daily saw this terrifying treatment scenario being added to the horrible effects of the disease. Yet they continued to work, until they succumbed. The mental health cost alone cannot be calculated. The burden of the illness, plus that of the treatment, was devastating.

## CLINICAL IMPRESSIONS

As a Physiatrist, my specialty is the assessment and treatment of impairment and disability. I estimate that I have assessed about 5000 third-party (employer, insurance company and WSIB of Ontario) disability cases. I have assessed and treated thousands of inpatients on rehabilitation units and in rehabilitation hospitals for impairment and disability.

The Long SARS patients were ordinary people, with no predisposing factors, who nearly drowned under a tsunami of severe illness and ongoing sequelae. This impression was echoed by almost everyone who came in contact with them. They had more of my empathy and sympathy than did any other patient group in my professional life.

Their physical rehabilitation was necessary. The exercises were very wearing, as fatigue was already one of their main symptoms. Their physical function did initially improve, but only slowly, with much effort required, and a disease-imposed cap on the outcome. If the improvement was small, why was it valuable? Because there was a very significant possibility that between deconditioning, weakness, fatigue, anxiety, depression and PTSD that these patients could have become even more limited in their function. So, although small gains were made in physical function, the primary benefit may have been to help prevent deterioration.

Professional psychological support was necessary for an extensive period of time. This was critical. In some ways, it was more important than were the physical treatments. In addition, if one facet of the program stood out as being highly valued by the patients, it was peer support.

I will point out that clinically none of our 50 patients got their old life back with time and treatment. Some were never able to return to work. Some had a trial of return to work and failed. Some had a trial of return to modified work, which then failed. Some had seniority to move to an easier position at work, which then failed. Some returned to being able to look after themselves completely, but could not return to work or sports. Some needed ongoing help (usually family) to do their daily activities. Some persisted in doing their daily activities, but slowly and intermittently, with frequent rests and dropping out of all non-essential activities. Not one reported that they were fully recovered and back to all their pre-SARS activities.

## DISCUSSION

Our hospital was involved during the spread of SARS pandemic in Toronto in 2003. In 2004, we were involved in a rehabilitation program for 50 health care workers disabled by their SARS illness. The patients had many, many ongoing symptoms, but the most prevalent were in the areas of fatigue, respiratory system, cognition, mental health and sleep disturbance. This is quite similar to the Long COVID patients’ symptoms as reported in the literature [8].

The multiple, frequent physical treatment interventions continued for a couple of years, but the patients were followed for much longer than that. Both my clinical observations and our incomplete data say that although there were early minor improvements, in the long term, there was incomplete resolution in terms of usual activities. The treatments possibly prevented deterioration even if they did not cause improvement. The patients remained physically impaired.

The patients had ongoing mental health impairments. Some required ongoing active psychological support, with data published for 7-year follow-up [7]. These symptoms were not improving and were showing no signs of eventual resolution. Cognitive issues were also proven [15]. Many mental health symptoms in Long Covid are also not resolving, but we have no truly long-term follow-up yet.

The lay press is frequently reporting on the numbers of people with symptoms of Long Covid and with questions about how long the symptoms will last. Some have already lasted for up to 2 years. Both the patients and their treating practitioners are concerned about the frequent failure of symptoms to resolve. The SARS-CoV-1 virus is similar to the SARS-CoV-2 virus. The two illnesses are similar. The ongoing symptoms resemble each other. Since we had cohort of Long SARS patients post serious illness, it seems logical to postulate that some of the Long Covid patients will also have permanent impairments. I fear that it has already happened and will continue to happen—although more time is needed to confirm this.

Covid outcomes are reported to range from no symptoms at all (asymptomatic) to death. Of the survivors, some will have Long Covid. Our Long SARS rehab program started with 50 patients—but they were pre-selected by WSIB to be the most seriously impaired. To compare, one has to postulate that there are Long Covid patients much less seriously impaired than were our patients. Those patients are likely to improve with time and preferentially with treatment. However, it seems likely that some patients will not only go on to Long Covid, but will also remain permanently impaired. This conclusion comes from both the data that we have and from my clinical impressions. It is not possible to predict this directly from a year or two of ongoing symptoms. It is possible to predict this by analogy to our Long SARS patients.

When Pandora opened her, box, she released sorrow, disease, vice, violence, greed, madness, old age and death into the world, and the only thing that remained to humans to battle these evils was Hope. Despite the likelihood that some people with Long Covid will have permanent impairments, we cannot deny them Hope. If hope is denied, efforts to mitigate will be abandoned by both the patients and their treating practitioners, creating outcomes that are worse than might otherwise be possible. There must be awareness that Long Covid symptoms may never fully resolve in some cases, but care must be taken to not take away all hope. Even prevention of deterioration can be worthwhile.

### And were lessons learned from the SARS pandemic?

I will not detail the efforts of the various levels of government to improve pandemic planning and to implement recommendations post SARS. I will let the history speak for itself. Absolutely, we were not prepared for the Covid pandemic. I have no patience with the argument that we have never seen anything like the Covid pandemic. We have indeed seen pandemics throughout history and must be better prepared for the next one. The next one is a certainty. The thing that is not known is the timing.

Highly recommended reading is the report of the SARS Commission [17]. Although it is longer than War and Peace [18], it is well worth the read. I strongly recommend reading the Executive Summary alone if the entire report is not to be read. Mr. Justice Archie Campbell of the Ontario Superior Court of Justice had a terrific gift for discarding confusion and nonsense. He cut directly to the heart of each episode and he wrote very well, concisely and logically. In fact, it reads much like a novel. As well as describing, the report made many, many recommendations. These were not directed as to how to medically treat sick patients, but were directed at all the institutions, great and small, that should be prepared to work together to limit future pandemics. Is the following recognizable today? From the Executive Summary, here are three paragraphs:

1. ‘Why was Ontario so unprepared for SARS? Our public health and emergency infrastructures were in a sorry state of decay, starved for resources by governments of all three political parties. The health system’s capacity to protect its workers was in a state of neglect: what little existed was badly malnourished. There was no system in place to prevent SARS or to stop it in its tracks. The only thing that saved us from a worse disaster was the courage and sacrifice and personal initiative of those who stepped up—the nurses, the doctors, the paramedics and all the others—sometimes at great personal risk, to get us through a crisis that never should have happened. Underlying all their work was the magnificent response of the public at large: patient, cooperative, supportive.’
2. ‘Why should we care about SARS now, 3 years after the event? We should care about SARS because we should never forget the loss and suffering, and we should never forget the courage shown by so many. We should care about SARS because it was a wake-up call and it holds the lessons we must learn to protect ourselves against future similar outbreaks and against the global influenza pandemic predicted by so many scientists’.
3. The most important lesson of SARS was implementing the ‘precautionary principle’, described as follows: ‘Where there is reasonable evidence of an impending threat to public harm, it is inappropriate to require proof of causation beyond a reasonable doubt before taking steps to avert the threat … that reasonable efforts to reduce risk need not await scientific proof’.

## TO CONCLUDE

It is clear that Long SARS (post SARS ongoing symptomatology) exists, persists (apparently permanently) and can be devastatingly life-changing for some. Sufficient similarities exist between Long SARS and Long Covid (PASC) in symptoms, findings and course over time (so far) that one can predict that it is very highly likely that some Long Covid disability will persist permanently. For those interested in rehabilitation, it is once more noted that the peer group support was very highly valued by the patients. Those wishing to treat Long Covid remotely (such as with telehealth) should consider this. All of the foregoing information is provided as a personal opinion to help guide treatment and counseling, so as to provide hope but not false hope for those affected by Long Covid.

## CONFLICT OF INTEREST STATEMENT

**None declared.**

## Appendix 1

**Table:**

Symptom	Current symptoms
Fatigue	1 2 3 4 5
Sleep disturbance	1 2 3 4 5
Depression	1 2 3 4 5
Anxiety	1 2 3 4 5
Anger	1 2 3 4 5
Memory problems	1 2 3 4 5
Concentration	1 2 3 4 5
Short of breath	1 2 3 4 5
Cough	1 2 3 4 5
Asthma	1 2 3 4 5
Wt. gain	1 2 3 4 5
Wt. loss	1 2 3 4 5
Hypertension	1 2 3 4 5
Pain: neck	1 2 3 4 5
Pain: back	1 2 3 4 5
Pain: arms	1 2 3 4 5
Pain: legs	1 2 3 4 5
Pain: head	1 2 3 4 5
Eyesight problems	1 2 3 4 5
